# Efficacy of high-fidelity simulation in advanced life support training: a systematic review and meta-analysis of randomized controlled trials

**DOI:** 10.1186/s12909-023-04654-x

**Published:** 2023-09-14

**Authors:** Qin Zeng, Kai Wang, Wei-xin Liu, Jiu-zhi Zeng, Xing-lan Li, Qing-feng Zhang, Shang-qing Ren, Wen-ming Xu

**Affiliations:** 1 Joint Laboratory of Reproductive Medicine, Key Laboratory of Obstetric, Gynecologic and Pediatric Diseases and Birth Defects of Ministry of Education, West China Second University Hospital, SCU-CUHK, Sichuan University, Chengdu, 610041 P. R. China; 2 Key Laboratory of Reproductive Medicine, Sichuan Provincial Maternity and Child Health Care Hospital, The Affiliated Women’s and children’s Hospital of Chengdu Medical College, Chengdu, 610045 China; 3 Department of Acute Care Surgery, Sichuan Provincial People’s Hospital, University of Electronic Science and Technology of China, Chengdu, 610072 China; 4 Department of pathology, Sichuan Provincial People’s Hospital, University of Electronic Science and Technology of China, Chengdu, 610072 China; 5 Ultrasound in Cardiac Electrophysiology and Biomechanics Key Laboratory of Sichuan Province, Sichuan Provincial People’s Hospital, School of Medicine, University of Electronic Science and Technology of China, Chengdu, 610072 China; 6 Robotic Minimally Invasive Surgery Center, Sichuan Provincial People’s Hospital, University of Electronic Science and Technology of China, Chengdu, 610072 China

**Keywords:** High-fidelity simulation, ALS, Skill performance, Meta-analysis, Participant’s confidence

## Abstract

**Background:**

Simulation is an increasingly used novel method for the education of medical professionals. This study aimed to systematically review the efficacy of high-fidelity (HF) simulation compared with low-fidelity (LF) simulation or no simulation in advanced life support (ALS) training.

**Methods:**

A comprehensive search of the PubMed, Chinese Biomedicine Database, Embase, CENTRAL, ISI, and China Knowledge Resource Integrated Database was performed to identify randomized controlled trials (RCTs) that evaluated the use of HF simulation in ALS training. Quality assessment was based on the Cochrane Handbook for Systematic Reviews of Interventions version 5.0.1. The primary outcome was the improvement of knowledge and skill performance. The secondary outcomes included the participants’ confidence and satisfaction at the course conclusion, skill performance at one year, skill performance in actual resuscitation, and patient outcomes. Data were synthesized using the RevMan 5.4 software.

**Results:**

Altogether, 25 RCTs with a total of 1,987 trainees were included in the meta-analysis. In the intervention group, 998 participants used HF manikins, whereas 989 participants received LF simulation-based or traditional training (classical training without simulation). Pooled data from the RCTs demonstrated a benefit in improvement of knowledge [standardized mean difference (SMD) = 0.38; 95% confidence interval (CI): 0.18–0.59, P = *0.0003*, *I*^2^ = 70%] and skill performance (SMD = 0.63; 95% CI: 0.21–1.04, P = 0.003, *I*^2^ = 92%) for HF simulation when compared with LF simulation and traditional training. The subgroup analysis revealed a greater benefit in knowledge with HF simulation compared with traditional training at the course conclusion (SMD = 0.51; 95% CI: 0.20–0.83, *P* = 0.003, *I*^2^ = 61%). Studies measuring knowledge at three months, skill performance at one year, teamwork behaviors, participants’ satisfaction and confidence demonstrated no significant benefit for HF simulation.

**Conclusions:**

Learners using HF simulation more significantly benefited from the ALS training in terms of knowledge and skill performance at the course conclusion. However, further research is necessary to enhance long-term retention of knowledge and skill in actual resuscitation and patient’s outcomes.

**Supplementary Information:**

The online version contains supplementary material available at 10.1186/s12909-023-04654-x.

## Background

Cardiovascular disease (CVD) accounts for 30% of all-cause mortality. On average, over 17.5 million people die of CVD annually worldwide, with approximately one death every 10 s [1]. Out-of-hospital cardiac arrest (OHCA) claims nearly 1.5 million lives annually [1]. High-quality cardiopulmonary resuscitation (CPR) is essential to successful OHCA. The American Heart Association (AHA) CPR Guidelines encourage closing the knowledge–practice gap and saving more lives [1]. However, a lack of practice remains a common complaint in medical education [2]. Simulation, as a novel method, is increasingly being used for the education of medical professionals. Simulation-based education has demonstrated many research benefits in improving skill performance, knowledge, and patient outcomes [3, 4]. High-fidelity (HF) manikins are widely used as part of the experiential learning component of advanced life-support (ALS) courses [5]. HF simulation provides real-time feedback on chest compression rate, depth, and recoil during advanced cardiac life support (ACLS) training and pediatric advanced life support (PALS) [5, 6].

Learners can assess physical findings and make clinical decisions regarding the simulated patients [5]. This may be ideal for providing opportunities for paramedics and medical students to practice their theoretical knowledge in simulated environments [7]. Studies have demonstrated the benefit of simulation training in various aspects of medical training [8, 9]. For ACLS training, the use of HF simulation has also been indicated to improve the knowledge translation of ACLS training [10–13]. Recent systematic reviews have revealed moderate benefits for improving skill performance in ALS training. However, research findings examining the benefit of HF simulation compared with low-fidelity (LF) simulation have yielded mixed findings. Several studies have demonstrated no distinct advantage of HF over LF simulations. Hence, teachers are perplexed by the conflicting nature of the evidence. HF is costly and time-consuming, as it requires specialized personnel, equipment, and space [14]. Therefore, a systematic review and meta-analysis to synthesize the current evidence is needed to assess the effectiveness of HF simulation in the learning process of ALS. This study aimed to identify the educational efficacy of HF simulation compared with no simulation or LF simulation in ALS training.

## Methods

This systematic review and meta-analysis was performed according to the Cochrane Reviewers’ Handbook and presented according to the Preferred Reporting Items for Systematic Reviews and Meta-analyses (PRISMA) guidelines. The protocol for this systematic review and meta-analysis is available at PROSPERO (CRD42022333898).

### Trial search

Randomized controlled trials (RCTs) were identified from PubMed, ISI (Web of Science: Science Citation Index Expanded), Cochrane Library (2022, Issue 8), China Knowledge Resource Integrated Database, World Health Organization Global Index Medicus, and Chinese Biomedicine Database from their inception dates to April 31, 2022. The search keywords and MESH terms were (“simulation” OR “patient simulation” OR “mannequin” OR “manikin”) AND (“life support care” OR “advanced life support” OR “neonatal resuscitation” OR “infant resuscitation”) AND (“education” OR “training” OR “teaching”). References from the RCTs were browsed, and the corresponding authors were consulted for any further information that they have not reported publicly. Ongoing RCTs were reviewed using clinical trial registers. The complete terms and strategies for identifying these articles are listed in the supplementary document (supplement 1).

### Inclusion and exclusion criteria

Only RCTs assessing the efficacy of HF simulation or manikins in ALS training in any language were included. Trials that did not address any of the primary or secondary outcomes were excluded. Intervention groups that received LF simulations and traditional training at any stage were accepted. All the participants were medical students and practitioners.

### Outcome measures

The primary outcome was the improvement in knowledge and skill performance. The secondary outcomes included the confidence of participants at the course conclusion, satisfaction of participants at the course conclusion, skill performance at one year; skill performance in resuscitation (compression rate, compression depth and compression fraction), teamwork behaviors and patient outcomes.

### Study selection and risk of bias assessments

The studies identified from the electronic searches were evaluated independently by 2 researchers (W.K. and Q.Z.) using a study eligibility form based on the inclusion criteria. Relevant studies were initially screened using titles and abstracts. Potential articles for inclusion were independently assessed by the two reviewers (W.K. and R.S.Q.), and any dissentions were resolved by a third reviewer (W.M.X.). The methodological quality of the included studies was assessed based on the Cochrane Reviewers’ Handbook [15]. Each study was evaluated for bias using the following items: randomization sequence generation, allocation concealment, blinding of participants and personnel, blinding of outcome assessment, incomplete outcome data, selective reporting, and other bias. If both allocation concealment and randomization had a low risk of bias and all other items had a low or unclear risk of bias, the trials were graded as high quality [16]. If either randomization or allocation concealment had a high risk of bias, the trials were considered of low quality, regardless of other items. If the trials did not meet the criteria of high or low risk of bias, they were graded as moderate quality.

### Data extraction and analysis

Data were independently extracted by two researchers (W.K. and Q.Z.) from the full text of the studies and compiled into shared sheets. The following data were collected from the included studies: study identifier (lead author and year of publication), country of origin, duration of ALS training, study design, inclusion and exclusion criteria, HF simulator type, number of participants, and primary and secondary outcomes. Only the data of interest were extracted when the trials had more than two group designs and permitted multiple comparisons. The data were validated by a third reviewer (W.M.X.) using a standardized method. In the case of inconclusive or missing data, the original authors were contacted to obtain missing details. RCTs reporting the same level of outcome were included in the quantitative synthesis. Risk ratio was used to report discrete numerical variables along with 95% confidence intervals (CIs). The standardized mean difference (SMD) was reported to estimate continuous outcomes. A fixed effect model was used when heterogeneity across studies was not detected. Otherwise, a random effect model was used, then the source of the heterogeneity would be analyzed by subgroup analysis. The *I*^2^ statistic was used to quantify heterogeneity, and forest plots were generated and counterchecked by two reviewers (S.Q.R. and W.M.X.). If *I*^2^ < 25%, the pooled outcomes were considered to have low statistical heterogeneity; if *I*^2^ > 75%, the pooled outcomes were considered to have high statistical heterogeneity. We also performed sensitivity analysis by the sequential removal of trials for each outcome. Publication bias was assessed by the funnel plots when no less than 10 trials were included in the meta-analysis. If data which were reported instead of mean and standard deviation (e.g., in case of median and range), we would transform it by the methods created by Hozo et al. and Higgins and Green. Data were synthesized using the RevMan 5.4 software. The research protocol, outcomes, and relevant items in this systematic review are reported following the PRISMA Statement [17].

## Results

### Included studies

**Fig. 1:**
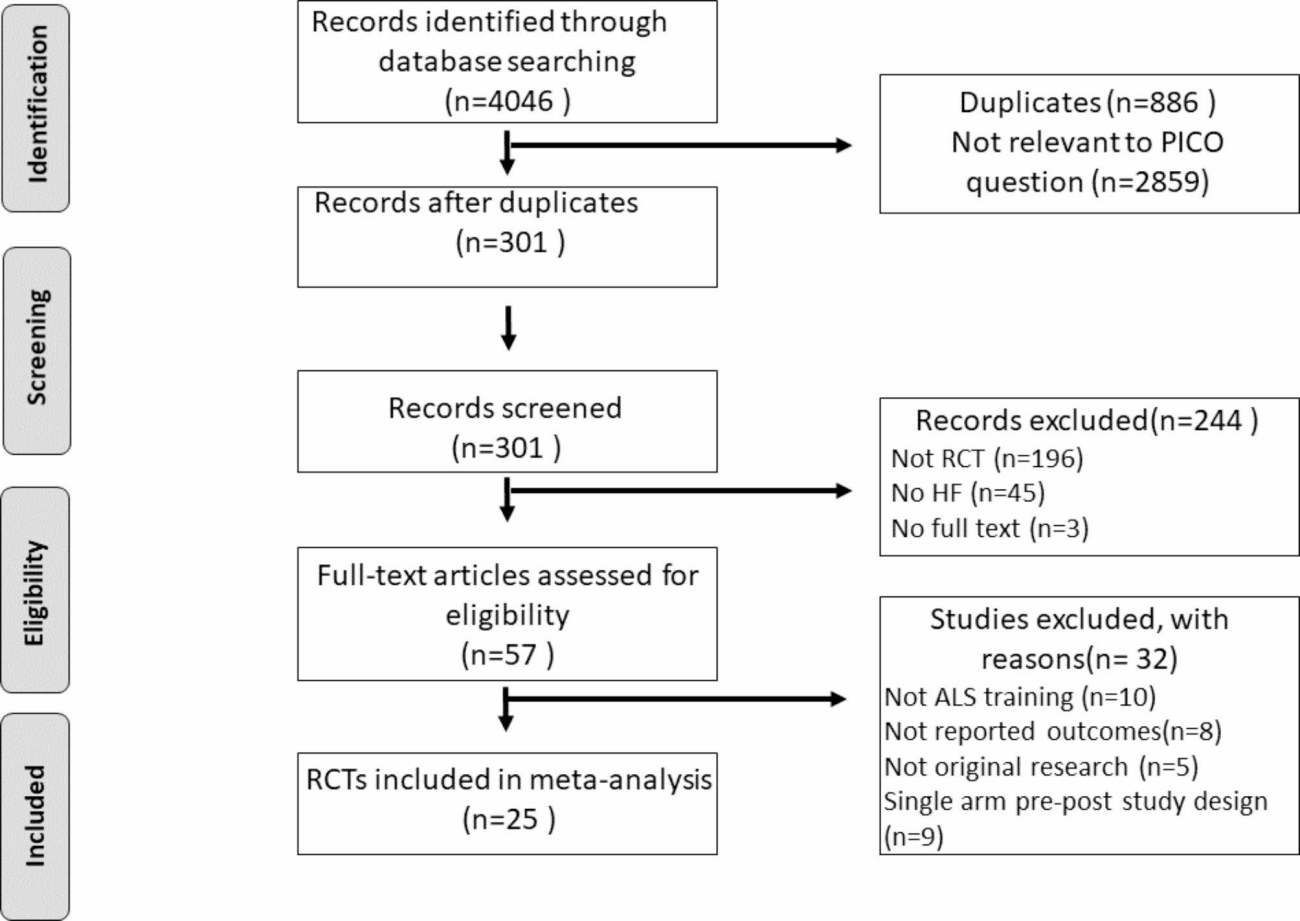
PRISMA diagram detailing the literature search and the study selection/exclusion process

A total of 4,046 studies were identified across databases in the initial literature review. Of these studies, 3,745 were excluded after reviewing the titles. The initial screening resulted in 57 candidate studies from a review of the abstracts. The reasons for exclusion are detailed in the PRISMA diagram (Fig. 1). In total, 25 RCTs [18–42] were identified for full analysis based on the inclusion and exclusion criteria. The design characteristics of the included studies are presented in Table 1. Of the included studies, eighteen studies compared HF simulation with LF simulation [18, 19, 21, 23, 25, 26, 28–37, 41, 42], and the other seven studies [20, 22, 24, 27, 38, 39, 42] compared traditional training. In total, 1,987 participants were included in the meta-analysis, of whom 998 were randomized to use HF manikins, whereas 989 received LF simulation-based or traditional training.

**Table 1:** Overview of the RCTs Included

Study	Simulation location	County	Participants	Context	Sample size	Results
Adams et al., 2015	University of Texas Health Sciences Center	America	First- and second-year medical students;First-year physician assistant students	ACLS	HF (n = 9) LF (n = 10)	No difference in knowledge and skill performance at course conclusion (P = 0.89).
Aqel et al., 2014	King Hussein Cancer Center in Amman	Jordan	Second-year nursing students	ACLS	HF (n = 45) LF (n = 45)	Improved knowledge and skill performance at course conclusion (P ≦ 0.01); and improved retention skill at 3 months after training (P ≦ 0.01).
Berger et al., 2019	General hospital ward	Germany	Fourth year medical students	ACLS	HF (n = 58) TT (n = 54)	Improved skill performance in the HF group at course conclusion (P = 0.007).
Campbell et al., 2009	St Michael’s Hospital	Canada	First year residents	NRP	HF(n = 8) LF (n = 7)	No difference in knowledge at course conclusion (p = 0.26).
Chen et al., 2015	The First Hospital Affiliated to AMU	China	medical students	NRP	HF (n = 20) TT (n = 20)	Improved knowledge at course conclusion (p < 0.05); and improved skill performance at course conclusion (p < 0.0001).
Cheng et al., 2013	Pediatric tertiary care centers	Canada	Allied health professionals, residents	PALS	HF (n = 197) LF (n = 190)	No difference in knowledge at course conclusion (p = 0.29); or skill performance at course conclusion (p = 0.23).
Cherry et al., 2007	The Pennsylvania State College of Medicine	America	First year residents	ATLS	HF (n = 23) TT (n = 21)	No difference in knowledge at course conclusion (p = 0.65); or skill performance at course conclusion (p = 0.23).
Conlon et al., 2014	Penn Medicine Clinical Simulation Center	America	Residents	ACLS	HF (n = 18) LF (n = 18)	Improved skill performance at the course conclusion in the HF group (P ≦ 0.04).
Coolen et al., 2012	Radboud University Nijmegen Medical Centre	The Netherlands	Fourth Year Medical Students	PALS	HF (n = 15) LF (n = 14)	No difference in knowledge at course conclusion (p = 0.4); and improved skill performance at course conclusion (p < 0.05).
Cortegiani et al., 2015	simulation centre of the University of Palermo	Italy	Fourth year medical students	ACLS	HF (n = 46) TT (n = 48)	Improved knowledge at course conclusion (P = 0.0017), although no difference in skill performance at course conclusion (P = 0.67).
Curran et al., 2015	Centre for Collaborative Health Professional Education	Canada	Third year undergraduate medical students	NRP	HF(n = 31) LF (n = 35)	No difference in skill performance at course conclusion (p = 0.45); but improved overall satisfaction (p = 0.001) and confidence (p = 0.001).
Donoghue et al., 2009	A.I. duPont Hospital for Children	Germany	Pediatric residents	PALS	HF(n = 25) LF (n = 26)	Improved skill performance at course conclusion in high fidelity group (p = 0.007).
Finan et al., 2012	University of Toronto	Canada	neonatal/perinatal fellowship trainees	NRP	HF(n = 16) LF (n = 16)	No difference in skill performance at course conclusion (p = 0.17).
Hoadley et al., 2009	OSF Saint Frances Medical Center College of Nursing	Frances	Physicians nurses respiratory therapists	ACLS	HF (n = 29) LF (n = 24)	No difference in knowledge at the course conclusion (P = 0.26); or skill performance at the course conclusion (P = 0.12).
King et al., 2011	Indiana University School of Nursing	America	Senior nursing students	ACLS	HF (n = 24) LF (n = 25)	No difference in knowledge at the course conclusion (P = 0.056).
Lo et al., 2011	Eastern Virginia Medical School	America	Medical students	ACLS	HF (n = 45) LF (n = 41)	Improved skill performance in the HF group (P < 0.0001). However, no difference in skill performance was observed at 1 year (P = 0.84)
Massoth et al., 2019	University Hospital Münster	Germany	Fourth-year medical students	ACLS	HF (n = 67) LF (n = 68)	Improved knowledge at course conclusion (P < 0.001).
McCoy et al., 2019	The UC Irvine Health Medical Education Simulation Center	America	Fourth-year medical students	ACLS	HF (n = 35) LF (n = 35)	Improved skill performance at the course conclusion (P = 0.02).
Nimbalkar et al., 2015	Pramukhswami Medical College	India	Undergraduate students	NRP	HF(n = 50) LF (n = 51)	No difference in knowledge at course conclusion (p = 0.38); or skill performance at course conclusion (p = 0.92).
Owen et al., 2006	Teaching hospitals in Adelaide	Australia	Interns and resident medical officers	ACLS	HF (n = 20) LF (n = 21)	Improved knowledge at the course conclusion (P = 0.026); but no difference in skill performance at the course conclusion (P = 0.084).
Rubio-Gurung et al., 2014	Croix-Rousse University Hospital	Frances	Level 1 and Level 2 maternities	NRP	HF (n = 6) TT (n = 6)	Improved skill performance (p = 0.004) and teamwork performance at course conclusion (p < 0.001).
Semler et al., 2015	The Center for Experiential Learning and Assessment facility at Vanderbilt University	America	Internal medicine interns	ACLS	HF(n = 17) TT (n = 18)	No difference in skill performance at course conclusion (p = 0.692); but improved teamwork performance at course conclusion (p = 0.045).
Settles et al., 2011	Indiana University School of Nursing	America	Healthcare students	ACLS	HF (n = 73) LF (n = 75)	No difference in knowledge at the course conclusion (P = 0.99); or skill performance at the course conclusion (P = 0.99).
Thomas et al., 2010	Surgical and Clinical Skills Center	America	residents	NRP	HF(n = 31) LF (n = 31)	No difference in skill performance at course conclusion (p = 0.654); but improved teamwork performance at course conclusion (p < 0.001).
Wang et al., 2017	Neonatal Diagnosis and Treatment Center	China	Medical students	NRP	HF (n = 90) TT (n = 90)	Improved knowlegde and skill performance at course conclusion (p < 0.001).

Abbreviations: HF, high-fidelity; LF, low-fidelity; ACLS, advanced cardiac life support; PALS, pediatric advanced life support; NRP, neonatal resuscitation program; TT, traditional training

### Participants and intervention

Of the 25 included studies, the participants included medical students (1,129), nursing students (90), residents (715), medical officers and paramedics (53). SimMan 3G by Laerdal is the most widely used simulator. All studies reported details of ALS training, which included the AHA course and group training using HF simulation cases. Twelve RCTs [18–20, 25, 27, 31–35, 37, 40] reported training duration and test time.

### Improvement of knowledge

**Fig. 2:**
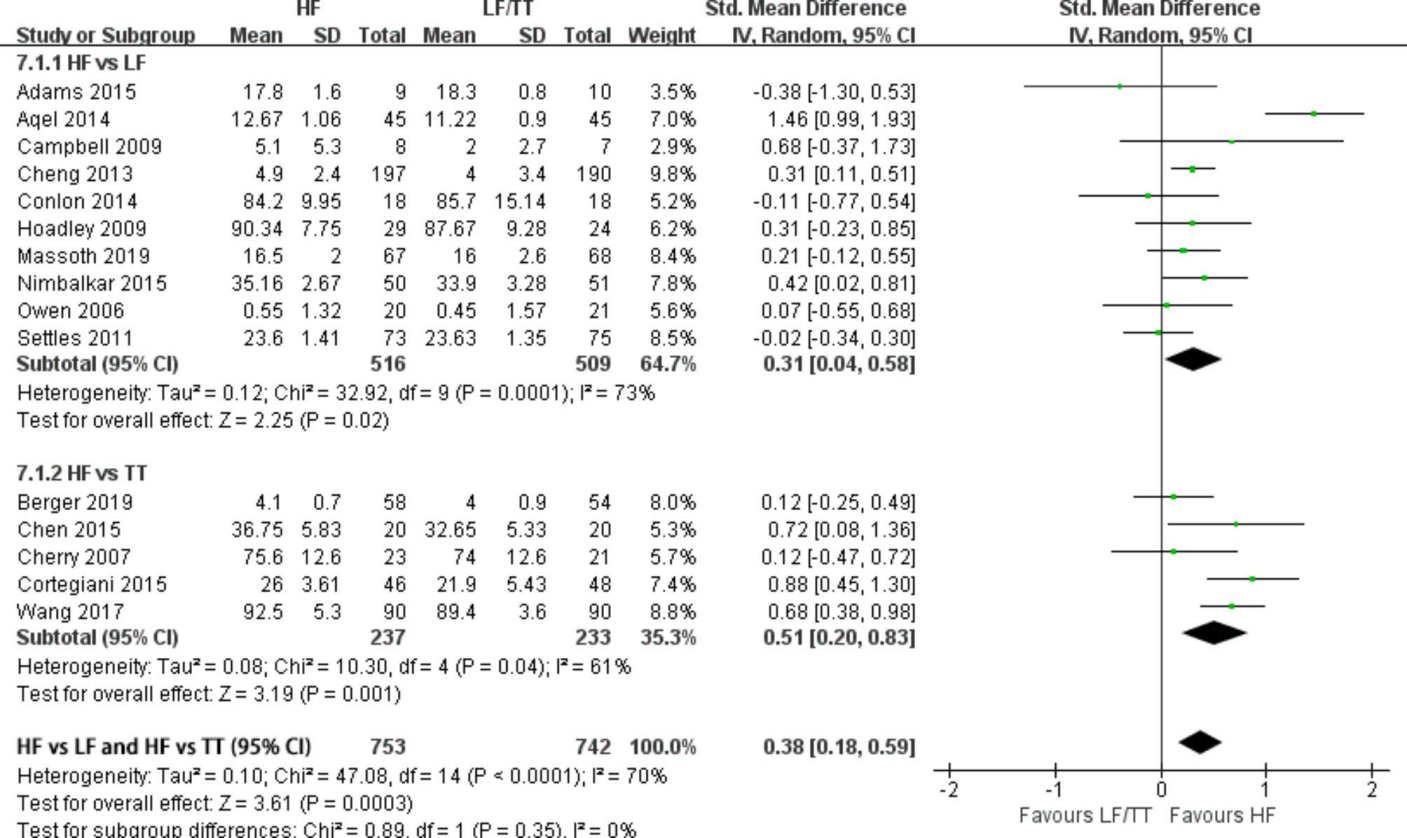
Forest plot of pooled weighted standardized mean difference from RCTs that evaluated the effects of improving knowledge with high-fidelity simulation at course conclusion. TT, traditional training

Fifteen studies [18–25, 27, 31, 34, 36, 37, 40, 42] reported data on knowledge measurement at the course conclusion. Pooled data from the RCTs demonstrated a benefit in improvement of knowledge for HF simulation when compared with LF simulation and traditional training (SMD = 0.38; 95% CI: 0.18–0.59, P = 0.0003, I^2^ = 70%]. (Fig. 2). I^2^ test discover significantly heterogeneity. Then we performed subgroup and sensitivity analyses, which found variation in course design, participants types, and outcomes measures in these studies [19, 20, 24] might be the source of heterogeneity. Subgroup analysis revealed that a benefit in knowledge with HF simulation compared with LF simulation (SMD = 0.22; 95% CI: 0.09–0.35, P = 0.001, I^2^ = 0%) and traditional training (SMD = 0.71; 95% CI: 0.51–0.97, P = 0.00001, I^2^ = 29%). (Supplement 2). Furthermore, we also performed a subgroup analysis depending on years of participation of the participant. The result show that residents (SMD = 0.28; 95% CI: 0.13–0.43, P = 0.0003, I^2^ = 0%) and three- and four-year medical/nurse students (SMD = 0.37; 95% CI: 0.04–0.69, P = 0.03, I^2^ = 77%) were benefit from HF simulation. However, fist-year medical/nurse students had no benefit from HF simulation (SMD = 0.66; 95% CI: − 0.31–1.64, P = 0.18, I^2^ = 85%). (Supplement 3). Four RCTs [19, 32, 36, 40] measured knowledge 3 months after training and have reported that both groups suffered a loss of knowledge (*P* < 0.001). However, no significant difference was observed between the HF and control groups (*P* = 0.28).

### Improvement of skill performance

**Fig. 3:**
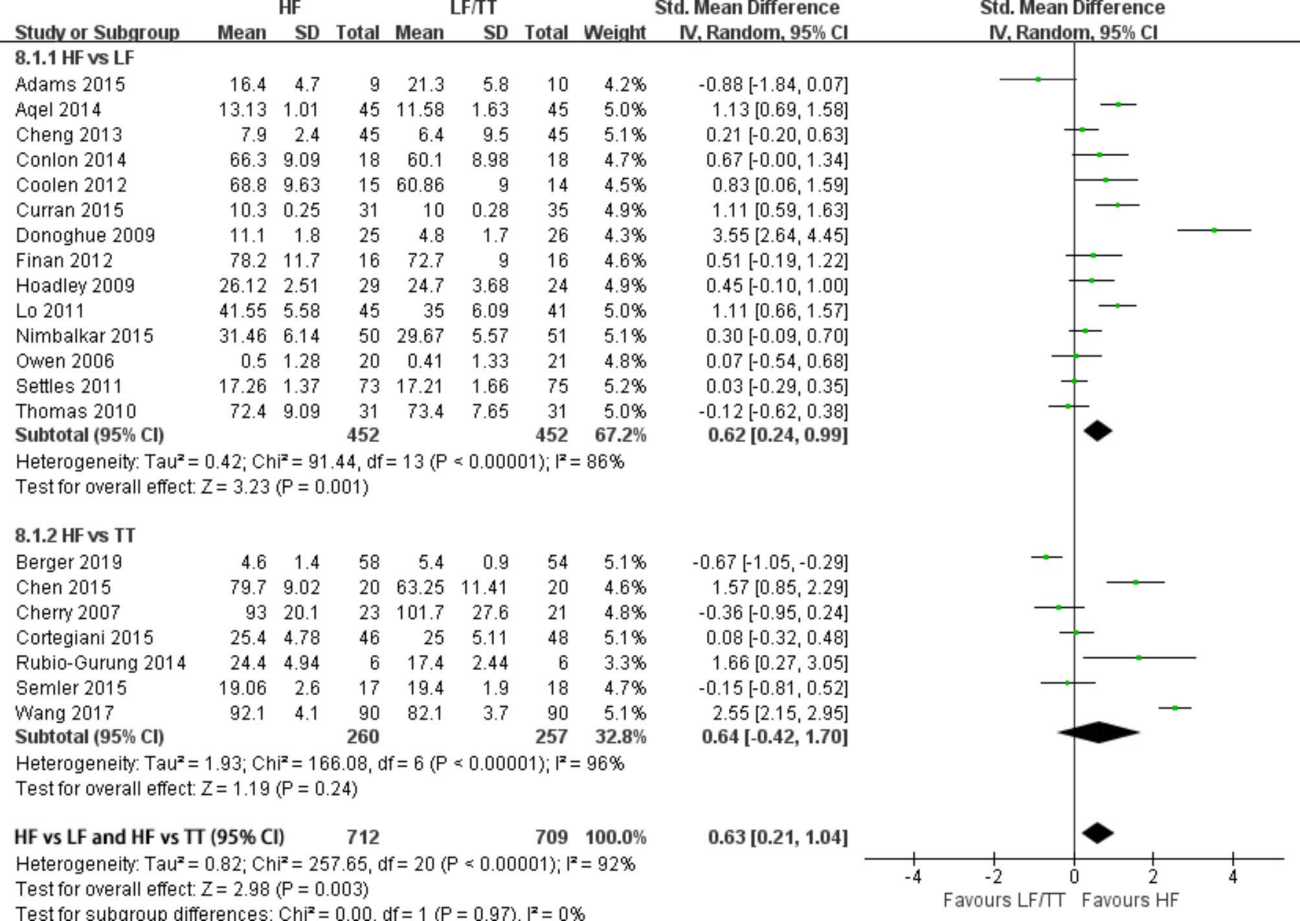
Forest plot of randomized controlled trials that evaluated the efficacy of high-fidelity simulation for improving skill performance at course conclusion. TT, traditional training

A total of twenty-one RCTs [18–20, 22–31, 33, 36–42] measured skill performance at the course conclusion. No significant difference was observed between the HF and control groups (SMD = 0.63; 95% CI: 0.21–1.04, P = 0.003, I^2^ = 92%). (Fig. 3) For high heterogeneity, we performed subgroup and sensitivity analyses, which found these studies [18–20, 22, 28, 29, 33, 39–42] with different training setting and outcomes measures might be the source of heterogeneity. In the subgroup analysis, a moderate benefit was observed for HF simulation compared with LF simulation at the course conclusion (SMD = 0.36; 95% CI: 0.16–0.57, *P* = 0.0004, *I*^2^ = 0%).(Supplement 4) However, no improvement in skill performance was observed in the HF group compared with those who received traditional training (SMD = -0.08; 95% CI: −0.37–0.22, *P* = 0.62, *I*^2^ = 0%). (Supplement 4). Furthermore, a subgroup analysis depending on years of participation of the participant also been conducted and found that only residents benefit from HF simulation in skill (SMD = 0.55; 95% CI: 0.05–1.05, *P* = 0.03, *I*^2^ = 86%). (Supplement 5). Only two RCTs [19, 40] measured skill 3 months after training and demonstrated that HF simulation was associated with moderate benefits for retaining ALS skills at 3 months after training (*P* ≤ 0.001).

### Confidence and satisfaction of participants

**Fig. 4:**
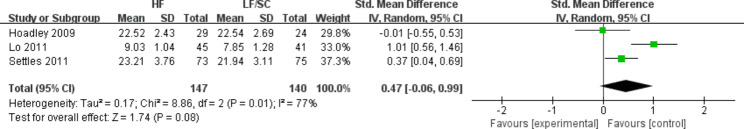
Random-effects meta-analysis of studies comparing HF simulation versus LF simulation or traditional training and reporting participants’ satisfaction

**Fig. 5:**
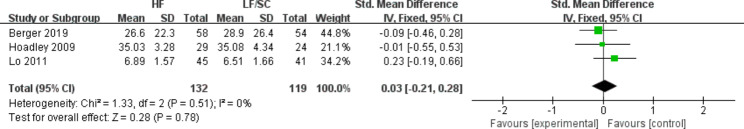
Fixed-effects meta-analysis of studies comparing HF simulation versus LF simulation or traditional training and reporting participants’ confidence

**Fig. 6:**
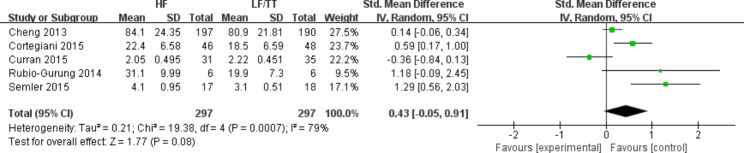
Random-effects meta-analysis of studies comparing HF simulation versus LF simulation or traditional training and reporting teamwork behaviors

Regarding the secondary outcomes, five studies [20, 28, 31, 33, 37] surveyed participants’ confidence, and three studies [31, 33, 40] reported participants’ satisfaction. Quantitative pooled data revealed that there was no difference in participants’ satisfaction between the HF and control groups (SMD = 0.47; 95% CI: -0.06–0.99, *P* = 0.08, *I*^2^ = 77%) (Fig. 4). Similarly, the confidence of the participants and teamwork behaviors in the HF group was not different from that of the control group (SMD = 0.03; 95% CI: −0.21–0.28, *P* = 0.78, *I*^2^ = 0%) (SMD = 0.43; 95% CI: −0.05–0.91, *P* = 0.08, *I*^2^ = 79%) (Figs. 5 and 6).

### Risk of bias within included studies

**Fig. 7:**
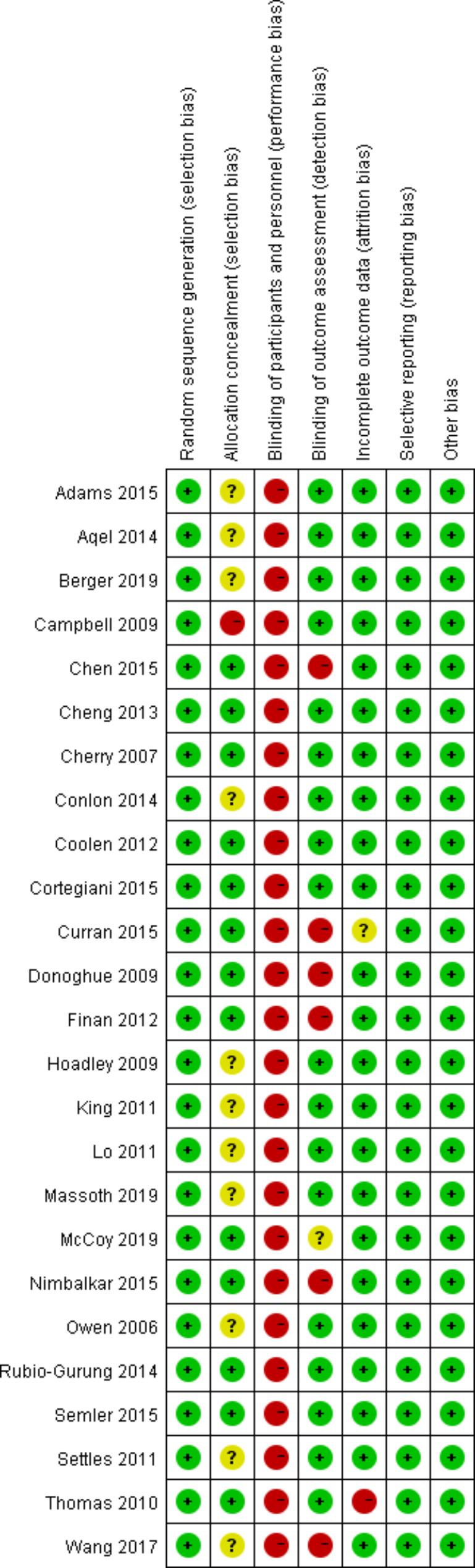
Risk of bias summary of the included randomized controlled trials. Green ‘+’ means low risk of bias, and yellow ‘?’ means unclear risk of bias

**Fig. 8:**
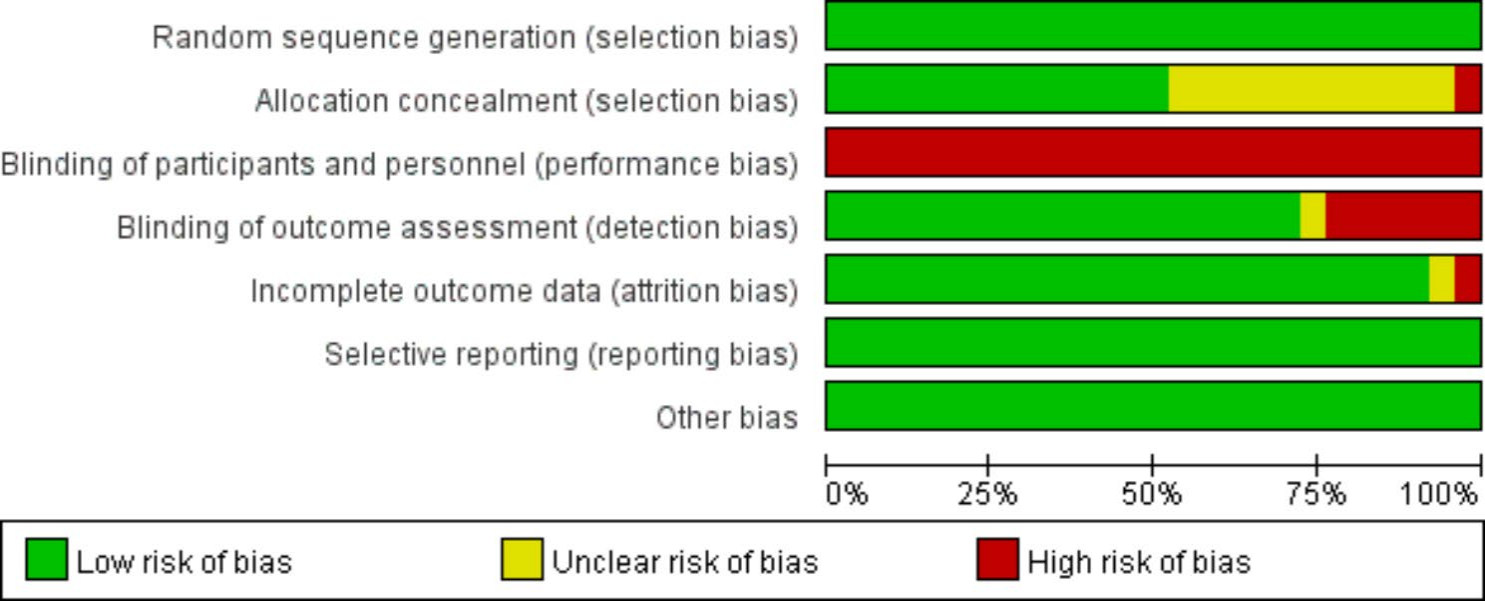
Risk of bias graph of the included randomized controlled trials

The risk of bias assessment of the included RCTs is summarized in Fig. 7. Performance bias existed in all RCTs because participant blinding to the level of fidelity is difficult to achieve. The concealment of 12 studies [18, 19, 25, 27, 31–35, 37, 40, 42] was unclear and incomplete. Meanwhile, two studies have reported moderate dropout rates [19, 33]. Finally, the risk of bias graph of the RCTs is presented in Fig. 8.

## Discussion

The results of this systematic review and meta-analysis indicated that participants benefited from improving knowledge and skill performance at the course conclusion with HF simulation. The subgroup analysis showed a greater benefit in knowledge with HF simulation compared with traditional training at the course conclusion. However, the use of HF simulation in comparison to LF simulation and traditional training showed no benefit for knowledge at 3 months, teamwork, and participant’s confidence. Higher means of satisfaction were observed in the HF group.

To the best of our knowledge, this is the first systematic review and meta-analysis of RCTs to identify the efficacy of HF simulation in ALS training. The benefits of HF simulation for improving skill performance and satisfaction in this study are consistent with the results of previous studies [1, 6, 43]. Cheng et al. compared HF manikins with LF manikins in ALS training [1]. They identified that HF manikins are moderately beneficial for improving skill performance at the conclusion of ALS training. In our study, the subgroup analysis also demonstrated a small to moderate benefit in skill performance for HF simulation when compare with LF simulation, but no benefit for HF simulation when compare with traditional training. A meta-analysis of 15 studies assessed the educational efficacy of simulations of a neonatal resuscitation program (NRP). The results revealed moderate effects favoring HF simulation for improving resuscitation knowledge and skill performance at the course conclusion [6]. Mundell et al. have reported that computer-controlled manikins are slightly beneficial for improving learner satisfaction and skills in ALS training [43]. A systematic review of emergency medicine training has demonstrated that technology-enhanced simulation is associated with greater benefits than traditional training [44]. These low-degree benefits may reveal the limitations of simulation, which is just a device that provides realistic feedback. A high efficacy ALS training also needs optimized course design, the case scenarios, experienced instructors and debriefing sessions. Different types of participants may not get the same benefit from HF simulation. The subgroup analysis of our study demonstrated that residents and three- and four-years medical/nurse students benefit from HF simulation, but first- and second-years medical/nurse students not. So, the high efficacy of HF simulation may be achieved in proper participants with well-design course, experienced instructors and debriefing in ALS training.

The retention of ALS knowledge and skills is widely recognized as a significant factor in actual resuscitation. Only two RCTs in this study [19, 40] have demonstrated that HF simulation was associated with moderate benefits for retaining ALS skills at 3 months after training. However, HF simulation did not significantly improve long-term retention of resuscitation knowledge [19, 32, 40]. Lack of retention might result from the quality of the content and the limitation of the training duration. Course duration and spaced practice are two key points for retaining knowledge and skill performance in the long term [43–47]. Wayne et al. have revealed that the use of eight hour of additional simulation training is associated with a greater benefit for retaining knowledge compared with none [9]. Evidence demonstrates that knowledge and skills deteriorate at 3 months after training course without ongoing practice [45]. The actual resuscitation performance of participants could be suboptimal during this interval. Efficacy of ALS training may be improved by increasing the frequency of HF simulation training, which may protect against knowledge and skill deterioration [46]. Additionally, spaced practice may improve the efficacy of training through elaborate learning and process information into a deeper memory. Studies found that after the initial training, repetition after a period of rest (weeks to months) better learning than practice massed within a very short period [47]. Therefore, further studies need to focus on improving the long-term retention of resuscitation knowledge and skills.

Teamwork is the key to resuscitation in a real clinical setting, and it could be improved through briefing and debriefing [48, 49]. During a debriefing, the leader should identify strengths and weaknesses in a positive forum, where everyone understands that the only goal is to enhance future performance. This step provides necessary feedback to team members and is crucial in enhancing team performance. Some studies have demonstrated that debriefing is associated with greater benefits for improving patient outcomes after cardiac arrest [50, 51]. Traditional training settings may not reflect the environment of actual team-based resuscitation. Since HF simulation-based training is often team-based, improving teamwork and debriefing may be more appropriate [33]. Previous studies have identified a small or moderate benefit for HF simulation-based training [42, 52]. One study has demonstrated that HF simulation improves teamwork performance in the NRP and PALS training [42]. Only five studies [27, 28, 33, 38, 39], including RCTs, assessed teamwork performance in ALS training. Pooled data from these RCTs demonstrated a trend benefit for improving teamwork behaviors for HF simulation when compared with LF simulation or traditional training. However, there were no significant difference in teamwork behaviors between HF simulation and control arm. Therefore, more high-quality RCTs are needed to assess the efficacy of HF simulation in improving teamwork performance. The best means of training debriefing sessions should be determined in order to enhance teamwork performance in HF simulation-based training.

While the goal of HF simulation-based training is to enhance knowledge and skills, cost-effectiveness is also a key point for educational intervention. If the cost of HF simulation-based training is prohibitively high, it could not be a viable option. Previous studies have analyzed the cost-effectiveness of simulation-based training programs on a learner basis [53]. Isaranuwatchai et al. conducted a cost-effectiveness analysis of HF and LF simulation training. The results of the study revealed that the HF program had the highest implementation cost [54]. At a willingness to pay of $100, HF programs had only a 6% probability of being cost-effective when compared with the LF program [54]. It is means that HF program was less cost-effective when compared to LF program. There was only a 6% chance that decision-makers were willing to pay $100 to buy HF program. None of the included RCTs assessed the cost-effectiveness of ALS training in HF simulations. Therefore, future studies should analyze the cost-effectiveness of HF simulation-based training.

### Limitations

This study had some limitations that need to be considered. First, of the included RCTs, no studies measured skill performance during actual resuscitation or patient outcomes. Furthermore, high heterogeneity was observed in a meta-analysis of knowledge and skill performance. Hence, the studies might have had different training settings, outcome measurements, and types of participants. Third, no uniform standard assessment of confidence and satisfaction was implemented. The included RCTs used various questionnaires, which may have led to unobjective and incomparable results. Finally, only few of the included studies met the high-quality standards of evidence-based medicine because the participants were not blinded to the level of simulation. We would suggest future work commit more resources to optimize instructional design, instructor and debriefing training.

## Conclusions

Learners using HF simulation benefited from knowledge and skill performance in ALS at the course conclusion than those using LF simulation or traditional training. A high-quality multicenter RCT is needed to enhance retention of knowledge and skills in actual resuscitation and patient outcomes in the future.

### Electronic supplementary material

Below is the link to the electronic supplementary material.

Supplementary Material 1

Supplementary Material 2

Supplementary Material 3

Supplementary Material 4

Supplementary Material 5

Supplementary Material 6

## Data Availability

All data generated or analyzed during this study are included in this published article [and its supplementary information files].

## Abbreviations

CBM: Chinese Biomedicine Database
CENTRAL: The Cochrane Library 2021, Issue 6
CNKI: China Knowledge Resource Integrated Database
ISI: Web of Science:Science Citation Index Expanded
PubMed: US National Library of Medicine

## References

[1] Cheng A, Lockey A, Bhanji F, Lin Y, Hunt EA, Lang E (2015). The use of high-fidelity manikins for advanced life support training–A systematic review and meta-analysis. Resuscitation.

[2] Roberts F, Cooper K (2019). Effectiveness of high fidelity simulation versus low fidelity simulation on practical/clinical skill development in pre-registration physiotherapy students: a systematic review. JBI Database System Rev Implement Rep.

[3] Dillon S (2021). Simulation in Obstetrics and Gynecology: a review of the past, Present, and Future. Obstet Gynecol Clin North Am.

[4] Weller J, Robinson B, Larsen P, Caldwell C (2004). Simulation-based training to improve acute care skills in medical undergraduates. N Z Med J.

[5] Ranger C, Paradis MR, Morris J, Perron R, Drolet P, Cournoyer A, Paquet J, Robitaille A (2018). Transcutaneous cardiac pacing competency among junior residents undergoing an ACLS course: impact of a modified high fidelity manikin. Adv Simul (Lond).

[6] Huang J, Tang Y, Tang J, Shi J, Wang H, Xiong T, Xia B, Zhang L, Qu Y, Mu D (2019). Educational efficacy of high-fidelity simulation in neonatal resuscitation training: a systematic review and meta-analysis. BMC Med Educ.

[7] Gardner R, Raemer DB (2008). Simulation in obstetrics and gynecology. Obstet Gynecol Clin North Am.

[8] Barsuk JHCE, McGaghie WC, Wayne DB (2010). Long-term retention of central venous catheter insertion skills after simulation-based mastery learning. Acad Med.

[9] Wayne DB, Butter J, Siddall VJ, Fudala MJ, Wade LD, Feinglass J, McGaghie WC (2006). Mastery learning of advanced cardiac life support skills by internal medicine residents using simulation technology and deliberate practice. J Gen Intern Med.

[10] Wayne DB, Siddall VJ, Butter J, Fudala MJ, Wade LD, Feinglass J, McGaghie WC (2006). A longitudinal study of internal medicine residents’ retention of advanced cardiac life support skills. Acad Med.

[11] Wayne DB, Didwania A, Feinglass J, Fudala MJ, Barsuk JH, McGaghie WC (2008). Simulation-based education improves quality of care during cardiac arrest team responses at an academic teaching hospital: a case-control study. Chest.

[12] Wayne DB, Butter J, Siddall VJ, Fudala MJ, Linquist LA, Feinglass J, Wade LD, McGaghie WC (2005). Simulation-based training of internal medicine residents in advanced cardiac life support protocols: a randomized trial. Teach Learn Med.

[13] Steadman RH, Coates WC, Huang YM, Matevosian R, Larmon BR, McCullough L, Ariel D (2006). Simulation-based training is superior to problem-based learning for the acquisition of critical assessment and management skills. Crit Care Med.

[14] Petscavage JM, Wang CL, Schopp JG, Paladin AM, Richardson ML, Bush WH (2011). Cost analysis and feasibility of high-fidelity simulation based radiology contrast reaction curriculum. Acad Radiol.

[15] Higgins JPT, Green S, editors. Cochrane handbook for systematic reviews of interventions version 5.0.1; updated September 2008. The Cochrane Collaboration. 2008; Available at: www.cochrane-handbook.org. Accessed October 15, 2009.

[16] Higgins JP, Altman DG, Gotzsche PC, Juni P, Moher D, Oxman AD, Savovic J, Schulz KF, Weeks L, Sterne JA (2011). The Cochrane collaboration’s tool for assessing risk of bias in randomised trials. BMJ.

[17] Page MJ, McKenzie JE, Bossuyt PM, Boutron I, Hoffmann TC, Mulrow CD, Shamseer L, Tetzlaff JM, Akl EA, Brennan SE (2021). The PRISMA 2020 statement: an updated guideline for reporting systematic reviews. BMJ.

[18] Adams AJ, Wasson EA, Admire JR, Pablo Gomez P, Babayeuski RA, Sako EY, Willis RE (2015). A comparison of Teaching Modalities and Fidelity of Simulation levels in teaching resuscitation scenarios. J Surg Educ.

[19] Aqel AA, Ahmad MM (2014). High-fidelity simulation effects on CPR knowledge, skills, acquisition, and retention in nursing students. Worldviews Evid Based Nurs.

[20] Berger C, Brinkrolf P, Ertmer C, Becker J, Friederichs H, Wenk M, Van Aken H, Hahnenkamp K (2019). Combination of problem-based learning with high-fidelity simulation in CPR training improves short and long-term CPR skills: a randomised single blinded trial. BMC Med Educ.

[21] Campbell DM, Barozzino T, Farrugia M, Sgro M (2009). High-fidelity simulation in neonatal resuscitation. Paediatr Child Health.

[22] Chen S, Wen C (2015). The applications of simbaby simulation training in the teaching of neonatal resuscitation. Chongqing Med J.

[23] Cheng A, Hunt EA, Donoghue A, Nelson-McMillan K, Nishisaki A, Leflore J, Eppich W, Moyer M, Brett-Fleegler M, Kleinman M (2013). Examining pediatric resuscitation education using simulation and scripted debriefing: a multicenter randomized trial. JAMA Pediatr.

[24] Cherry RA, Williams J, George J, Ali J (2007). The effectiveness of a human patient simulator in the ATLS shock skills station. J Surg Res.

[25] Conlon LW, Rodgers DL, Shofer FS, Lipschik GY (2014). Impact of levels of simulation fidelity on training of interns in ACLS. Hosp Pract.

[26] Coolen EH, Draaisma JM, Hogeveen M, Antonius TA, Lommen CM, Loeffen JL. Effectiveness of high fidelity video-assisted real-time simulation: a comparison of three training methods for acute pediatric emergencies. Int J Pediatr. 2012; 2012:709569. 10.1155/2012/709569 PMC3299281 22518181

[27] Cortegiani A, Russotto V, Montalto F, Iozzo P, Palmeri C, Raineri SM, Giarratano A (2015). Effect of High-Fidelity Simulation on Medical students’ knowledge about Advanced Life support: a randomized study. PLoS ONE.

[28] Curran V, Fleet L, White S, Bessell C, Deshpandey A, Drover A, Hayward M, Valcour J (2015). A randomized controlled study of manikin simulator fidelity on neonatal resuscitation program learning outcomes. Adv Health Sci Educ Theory Pract.

[29] Donoghue AJ, Durbin DR, Nadel FM, Stryjewski GR, Kost SI, Nadkarni VM (2009). Effect of high-fidelity simulation on Pediatric Advanced Life Support training in pediatric house staff: a randomized trial. Pediatr Emerg Care.

[30] Finan E, Bismilla Z, Whyte HE, Leblanc V, McNamara PJ (2012). High-fidelity simulator technology may not be superior to traditional low-fidelity equipment for neonatal resuscitation training. J Perinatol.

[31] Hoadley TA (2009). Learning advanced cardiac life support: a comparison study of the effects of low- and high-fidelity simulation. Nurs Educ Perspect.

[32] King JM, Reising DL (2011). Teaching advanced cardiac life support protocols: the effectiveness of static versus high-fidelity simulation. Nurse Educ.

[33] Lo BM, Devine AS, Evans DP, Byars DV, Lamm OY, Lee RJ, Lowe SM, Walker LL (2011). Comparison of traditional versus high-fidelity simulation in the retention of ACLS knowledge. Resuscitation.

[34] Massoth C, Röder H, Ohlenburg H, Hessler M, Zarbock A, Pöpping DM, Wenk M. High-fidelity is not superior to low-fidelity simulation but leads to overconfidence in medical students. BMC Med Educ. 2019; 19(29). 10.1186/s12909-019-1464-7 PMC6341720 30665397

[35] McCoy CE, Rahman A, Rendon JC, Anderson CL, Langdorf MI, Lotfipour S, Chakravarthy B (2019). Randomized Controlled Trial of Simulation vs. Standard Training for Teaching Medical Students High-quality cardiopulmonary resuscitation. West J Emerg Med.

[36] Nimbalkar A, Patel D, Kungwani A, Phatak A, Vasa R, Nimbalkar S (2015). Randomized control trial of high fidelity vs low fidelity simulation for training undergraduate students in neonatal resuscitation. BMC Res Notes.

[37] Owen H, Mugford B, Follows V, Plummer JL (2006). Comparison of three simulation-based training methods for management of medical emergencies. Resuscitation.

[38] Rubio-Gurung SPG, Touzet S, Gauthier-Moulinier H, Jordan I, Beissel A, Labaune JM, Blanc S, Amamra N, Balandras C, Rudigoz RC (2014). In situ simulation training for neonatal resuscitation: an RCT. Pediatrics.

[39] Rubio-Gurung S, Putet G, Touzet S, Gauthier-Moulinier H, Jordan I, Beissel A, Labaune JM, Blanc S, Amamra N, Balandras C (2015). A randomized trial comparing didactics, demonstration, and simulation for teaching teamwork to medical residents. Ann Am Thorac Soc.

[40] Settles J, Jeffries PR, Smith TM, Meyers JS (2011). Advanced cardiac life support instruction: do we know tomorrow what we know today?. J Contin Educ Nurs.

[41] Thomas EJ, Williams AL, Reichman EF, Lasky RE, Crandell S, Taggart WR (2010). Team training in the neonatal resuscitation program for interns: teamwork and quality of resuscitations. Pediatrics.

[42] Wang JH, Zhang XH, Fan J (2017). An application study of simulating teaching method on neonatal resuscitation training for medical students. China Contin Med Educ.

[43] Mundell WC, Kennedy CC, Szostek JH, Cook DA (2013). Simulation technology for resuscitation training: a systematic review and meta-analysis. Resuscitation.

[44] Ilgen JS, Sherbino J, Cook DA (2013). Technology-enhanced simulation in emergency medicine: a systematic review and meta-analysis. Acad Emerg Med.

[45] Cheng A, Nadkarni VM, Mancini MB, Hunt EA, Sinz EH, Merchant RM, Donoghue A, Duff JP, Eppich W, Auerbach M (2018). Resuscitation Education Science: Educational Strategies to improve outcomes from Cardiac arrest: a Scientific Statement from the American Heart Association. Circulation.

[46] Anderson R, Sebaldt A, Lin Y, Cheng A (2019). Optimal training frequency for acquisition and retention of high-quality CPR skills: a randomized trial. Resuscitation.

[47] Lin Y, Cheng A, Grant VJ, Currie GR, Hecker KG (2018). Improving CPR quality with distributed practice and real-time feedback in pediatric healthcare providers - a randomized controlled trial. Resuscitation.

[48] Brogaard L, Hvidman L, Esberg G, Finer N, Hjorth-Hansen KR, Manser T, Kierkegaard O, Uldbjerg N, Henriksen TB (2022). Teamwork and adherence to Guideline on Newborn Resuscitation-Video review of neonatal interdisciplinary teams. Front Pediatr.

[49] Hunziker S, Johansson AC, Tschan F, Semmer NK, Rock L, Howell MD, Marsch S (2011). Teamwork and leadership in cardiopulmonary resuscitation. J Am Coll Cardiol.

[50] Edelson DP, Litzinger B, Arora V, Walsh D, Kim S, Lauderdale DS, Vanden Hoek TL, Becker LB, Abella BS (2008). Improving in-hospital cardiac arrest process and outcomes with performance debriefing. Arch Intern Med.

[51] Wolfe H, Zebuhr C, Topjian AA, Nishisaki A, Niles DE, Meaney PA, Boyle L, Giordano RT, Davis D, Priestley M (2014). Interdisciplinary ICU cardiac arrest debriefing improves survival outcomes. Crit Care Med.

[52] Sawyer T, Sierocka-Castaneda A, Chan D, Berg B, Lustik M, Thompson M (2011). Deliberate practice using simulation improves neonatal resuscitation performance. Simul Healthc.

[53] Scott DJ, Goova MT, Tesfay ST (2007). A cost-effective proficiency-based knot-tying and suturing curriculum for residency programs. J Surg Res.

[54] Isaranuwatchai W, Brydges R, Carnahan H, Backstein D, Dubrowski A (2014). Comparing the cost-effectiveness of simulation modalities: a case study of peripheral intravenous catheterization training. Adv Health Sci Educ Theory Pract.

