# Supplementary material for: Efficacy of high-fidelity simulation in advanced life support training: a systematic review and meta-analysis of randomized controlled trials
Source: BMC Med Educ. 2023 Sep 14;23:664. doi: 10.1186/s12909-023-04654-x (PMC10500810; doi:10.1186/s12909-023-04654-x)
Supplement: Supplementary file 1 — Supplementary Material 1 [file 12909_2023_4654_MOESM1_ESM.docx]

| Supplementary 1: Search Terms and Complete Strategy | | |
| --- | --- | --- |
| Database | Search Terms | Search Strategy |
| PubMed | 1. (("Simulation Training"[Mesh]) OR (((Training, Simulation) OR (Interactive Learning)) OR (Learning, Interactive))) OR (("Manikins"[Mesh]) OR (((Manikin) OR (Mannequins)) OR (Mannequin)))  2. ((("Cardiovascular Diseases"[Mesh]) OR ((((Cardiovascular Disease) OR (Disease, Cardiovascular)) OR (Diseases, Cardiovascular)) OR (cardiac arrest))) OR (("Heart Arrest"[Mesh]) OR (((((((Arrest, Heart) OR (Cardiac Arrest)) OR (Arrest, Cardiac)) OR (Asystole)) OR (Asystoles)) OR (Cardiopulmonary Arrest)) OR (Arrest, Cardiopulmonary)))) AND (("Advanced Cardiac Life Support"[Mesh]) OR ((Cardiac Life Support, Advanced) OR (Life Support, Advanced Cardiac)) OR ((neonatal resuscitation) OR (infant resuscitation)))  3.((randomized controlled trial[pt] OR controlled clinical trial[pt]) OR (“Clinical Trials as Topic”[MeSH Major Topic])) NOT ((“Animals”[Mesh]) NOT (“Humans”[Mesh] AND “Animals”[Mesh])) | #1 AND #2 AND #3 |
| Embase | #1. 'training, simulation' OR (('training,'/exp OR training,) AND ('simulation'/exp OR simulation)) OR (simulation AND training) OR (training, AND simulation) OR (interactive AND learning) OR (learning, AND interactive)  #2. 'manikins'/exp OR manikins OR manikin OR mannequins OR mannequin  #3. 'heart arrest'/exp OR 'heart arrest' OR (('heart'/exp OR heart) AND ('arrest'/exp OR arrest)) OR (arrest, AND heart) OR (cardiac AND arrest) OR (arrest, AND cardiac) OR asystole OR asystoles OR (cardiopulmonary AND arrest) OR (arrest, AND cardiopulmonary) OR  #4. 'cardiovascular disease'/exp OR 'cardiovascular disease' OR (('cardiovascular'/exp OR cardiovascular) AND ('disease'/exp OR disease)) OR (cardiovascular AND disease) OR (disease, AND cardiovascular) OR (diseases, AND cardiovascular)  #5. 'advanced cardiac life support'/exp OR 'advanced cardiac life support' OR (advanced AND ('cardiac'/exp OR cardiac) AND ('life'/exp OR life) AND ('support'/exp OR support)) OR (cardiac AND life AND support, AND advanced) OR (life AND support, AND advanced AND cardiac) OR (neonatal resuscitation) OR (infant resuscitation) | (#1 OR #2) AND (#3 OR #4) AND #5 |
| ISI | 1.Topic=(Simulation Training OR Interactive Learning OR high-fidelity simulation OR low-fidelity simulation OR Manikins OR Mannequins OR Mannequin)  2.Topic=(Cardiovascular Diseases OR Cardiac Arrest OR Heart Arrest OR) AND (Advanced Cardiac Life Support OR Cardiac Life Support OR Life Support OR neonatal resuscitation OR infant resuscitation)  3.TS=(clinical OR control OR random OR placebo OR randomised OR randomized OR randomly OR random order OR random sequence OR random allocation OR randomly allocated OR at random) | 1 and 2 and 3 |
| Chchrane | 1.(simulation training or interactive learning or high-fidelity simulation or low-fidelity simulation or manikins or mannequin or mannequins)  2.(cardiovascular diseases or cardiovascular disease or cardiac arrest or heart arrest)  3.(advanced cardiac life support or cardiac life support or life support) | 1 AND 2 AND 3 |
| CBM | 1. "simulation Training "[ Full field] 2. " interactive learning "[ MeSH]) 3. "high-fidelity simulation"[ Full field] 4. "low-fidelity simulation"[ MeSH] 5. "manikins"[ Full field] 6. "mannequin"[ Full field] 7. "manikins"[ Full field] 8. "cardiovascular diseases"[ Full field] 9. "cardiac arrest"[ Full field] 10. "advanced cardiac life support"[ Full field] 11. "cardiac life support"[ MeSH] 12. "neonatal resuscitation"[ Full field] 13 "infant resuscitation" [ Full field] | (1 OR 2 OR 3 OR 4 OR 5 OR 6 OR 7) AND (8 OR 9) AND (10 OR 11 OR 12 OR 13) |
| CNKI | 1.KY= simulation training 2.KY= interactive learning 3.KY= manikins 4. KY= cardiovascular diseases 5.KY= cardiac arrest 6.KY= advanced cardiac life support 7.KY= neonatal resuscitation 8.KY= infant resuscitation | (1 OR 2 OR 3) AND (4 OR 5) AND (6 OR 7 OR 8) |
