# Supplementary material for: Efficacy of high-fidelity simulation in advanced life support training: a systematic review and meta-analysis of randomized controlled trials
Source: BMC Med Educ. 2023 Sep 14;23:664. doi: 10.1186/s12909-023-04654-x (PMC10500810; doi:10.1186/s12909-023-04654-x)
Supplement: Supplementary file 2 — Supplementary Material 2 [file 12909_2023_4654_MOESM2_ESM.docx]

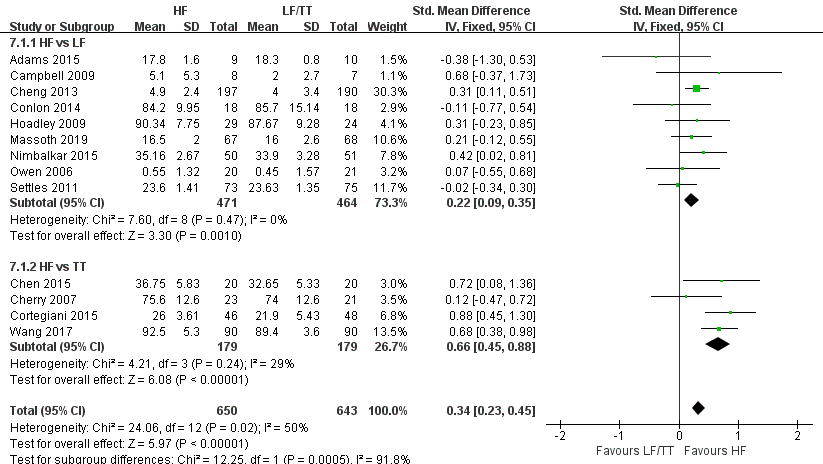


Supplement 2: Sensitivity analyses revealed that two studies might be the source of heterogeneity. Forest plot of pooled weighted standardized mean difference from remaining RCTs that evaluated the effects of improving knowledge with high-fidelity simulation at course conclusion.
