# Supplementary material for: Efficacy of high-fidelity simulation in advanced life support training: a systematic review and meta-analysis of randomized controlled trials
Source: BMC Med Educ. 2023 Sep 14;23:664. doi: 10.1186/s12909-023-04654-x (PMC10500810; doi:10.1186/s12909-023-04654-x)
Supplement: Supplementary file 5 — Supplementary Material 5 [file 12909_2023_4654_MOESM5_ESM.docx]

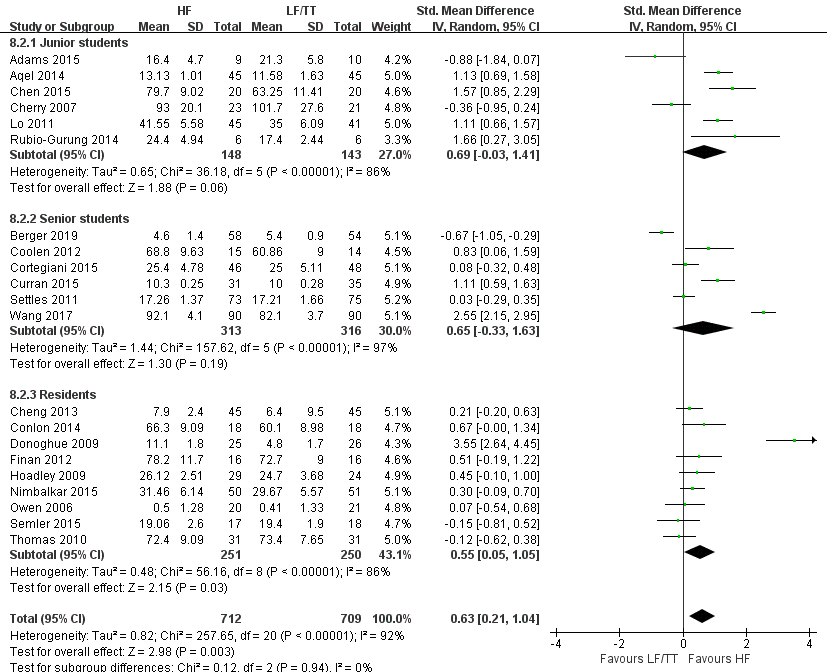


Supplement 5: Subgroup analysis: effects of improving skill performance with high-fidelity simulation at course conclusion.
